# *Pseudocohnilembus persalinus* genome database - the first genome database of facultative scuticociliatosis pathogens

**DOI:** 10.1186/s12864-018-5046-6

**Published:** 2018-09-14

**Authors:** Wei Wei, Kai Chen, Wei Miao, Wentao Yang, Jie Xiong

**Affiliations:** 1 Key Laboratory of Aquatic Biodiversity and Conservation, Institute of Hydrobiology, Chinese Academy of Sciences, Wuhan, 430072 China; 2 University of Chinese Academy of Sciences, Beijing, 100049 China

**Keywords:** *Pseudocohnilembus persalinus*, Genome database, Useful resource, Scuticociliate

## Abstract

**Background:**

*Pseudocohnilembus persalinus*, a unicellular ciliated protozoan, is one of commonest facultative pathogens. We sequenced the macronuclear genome of *P. persalinus* in 2015, which provided new insights into its pathogenicity.

**Results:**

Here, we present the *P. persalinus* genome database (PPGD) (http://ciliates.ihb.ac.cn/database/home/#pp), the first genome database for the scuticociliatosis pathogens. PPGD integrates *P. persalinus* macronuclear genomic and transcriptomic data, including genome sequence, transcript, gene expression data, and gene annotation, as well as relevant information on its biology, morphology and taxonomy. The database also provides functions for visualizing, analyzing, and downloading the data.

**Conclusion:**

PPGD is a useful resource for studying scuticociliates or scuticociliatosis. We will continue to update the PPGD by integrating more data and aim to integrate the PPGD with other ciliate databases to build a comprehensive ciliate genome database.

## Background

The *Pseudocohnilembus persalinus* scuticociliate is a free-living marine ciliate first reported by Evans and Thompson in 1964 [1]. Since then, its morphological, ecological, and phylogenetic have been well studied [2–4]. Since Kim and colleagues (2004) isolated *P. persalinus* from a diseased olive flounder in Korea, the organism has been recognized as a common facultative pathogen. *P. persalinus* infection causes scuticociliatosis, one of the most important fish diseases that has led to serious economic losses in marine aquaculture worldwide [5, 6]. *P. persalinus* is a facultative parasite and can be easily grown in the laboratory by feeding with bacteria. It is therefore a suitable model for studying the life cycle, genetics, and genomics of ciliates. Therefore, *P. persalinus* is an ideal model for investigating scuticociliatosis and the molecular mechanisms of its pathogenicity.

Like other ciliates, it has two types of functionally diverse nuclei within the same cytoplasm: a micronucleus (MIC) and a macronucleus (MAC). The MIC is transcriptionally silent and undergoes meiosis and transmits the genetic information to the progeny by sexual reproduction. In contrast, the MAC is highly polyploid and transcriptionally active, and controls the non-reproductive features of cell function. In 2015, we reported the *P. persalinus* MAC genome sequence [7]. This was the first report of its type for a scuticociliate and a marine ciliate. Before that, the genomes of *Tetrahymena thermophila*, *Paramecium tetraurelia* and *Ichthyophthirius multifiliis* have first been sequenced and their comparative genomics analyses have comprehensively provided a better understanding of the unique features between the free-living (*T. thermophila* and *P. tetraurelia*) and typical parasitic (*I. multifiliis*) ciliates. As has been done for these ciliates, we have constructed the *P. persalinus* genome database (PPGD), which integrates genomic data, transcriptomic data, and gene annotation, thereby providing a useful resource for studying scuticociliates or scuticociliatosis.

## Construction

**Fig. 1:**
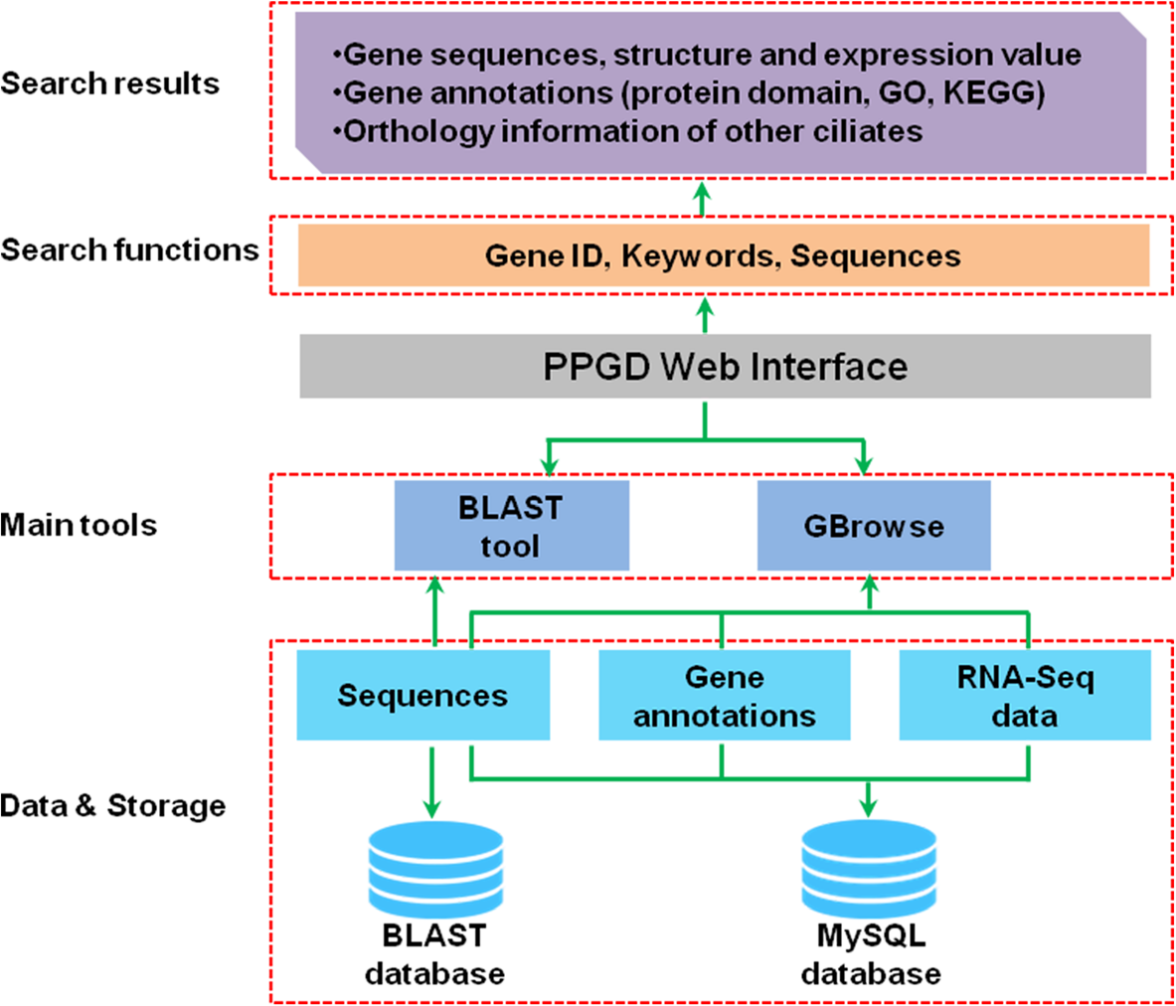
Diagram of the PPGD. The sequence data was formatted and incorporated into the BLAST web service. Sequence data, gene annotation, and RNA-Seq data were stored in the MySQL database, and also presented by GBrowse. Meanwhile, search functions enable the user to easily access the resources in PPGD

Figure 1 shows a schematic diagram of the structure of the PPGD. Genomic sequence data, transcriptomic sequence (RNA-Seq) data, and genome annotation data are stored in the MySQL database. To use BLAST tool, genomic sequence was also formatted as BLAST database. All data are freely available for downloading. The user-friendly interface and web pages were developed with PHP and HTML. The entire PPGD runs on an Apache HTTP server and uses MySQL as the database management system. In addition, the Generic Genome Browser (GBrowse) [8], a mature, widely used genome browser, was incorporated to manipulate and visualize genome annotation and expression data. Online analysis tools in PPGD were implemented with in-house PHP scripts or by wrapping existing third-party tools (e.g. BLAST server).

## Content

PPGD mainly contains three types of datasets containing: (i) genomic sequence; (ii) gene expression data; and (iii) genome annotation. The PPGD also includes a brief summary of the biological, morphological, and taxonomical characteristics of *P. persalinus*.

The *P. persalinus* genome was sequenced with the Illumina platform and then assembled with SOAPdenovo [9]. The 55.5 Mb genome was assembled from 288 scaffolds; the scaffold N50 is approximately 368 kb [7]. A total of 13,186 genes were predicted by an ab initio prediction pipeline. Three types of annotation were included for these genes. The first type was functional prediction based on the BLAST hits when searched against the NCBI non-redundant protein database. The function of 4,265 genes (32%) could be assigned in this way; all other genes were annotated as “hypothetical protein”. The second type was protein domain or structure annotation based on InterProScan prediction [10]. InterProScan integrates the results from multiple protein domain databases (Pfam, PRINTS, PANTHER, Gene3D and InterProScan) and some protein domain structures (coiled-coil, signal peptide, and transmembrane helix). A total of 11,948 genes could be annotated and 5,869 genes could be assigned GO (gene ontology) numbers based on InterProScan predictions. The third type was based on gene information on homologs in other ciliates. Ortholog groups in six ciliates (*Ichthyophthirius multifiliis*, *Oxytricha trifallax*, *Paramecium tetraurelia*, *P. persalinus*, *Stylonychia lemnae*, and *Tetrahymena thermophila*) were obtained using OrthoMCL [11], finally, 5,962 *P. persalinus* genes could be assigned, and thus provided in PPGD. We also used RNA-Seq data to obtain gene expression data for *P. persalinus* fed with bacteria to determine changes in gene transcription level under growth condition. The RNA-seq data was mapped to the *P. persalinus* MAC assembly using TopHat2 [12], then raw read counts were calculated for each gene using Subread [13] program, and the gene expression values were obtained by normalized the raw read counts to RPKM (reads per kilobase per million mapped reads) values. For all the 13,186 predicted genes, 92% (12,145) of genes have more or less reads mapped in the growth condition. Among them, 56% (6765) of genes showed an expression value larger than 10.

## Utility and discussion

### Search functions

PPGD can be accessed via an easy-to-use web interface. The top row of navigation tabs (including “HOME,” “SEARCH,” “GENOME BROWSER,” “BLAST,” “MORPHOLOGY,” and “DATA DOWNLOAD”) directs the user to retrieve the information. The “HOME” page displays some background information on *P. persalinus*, introduces the database, and contains news and references related to the PPGD.

**Fig. 2:**
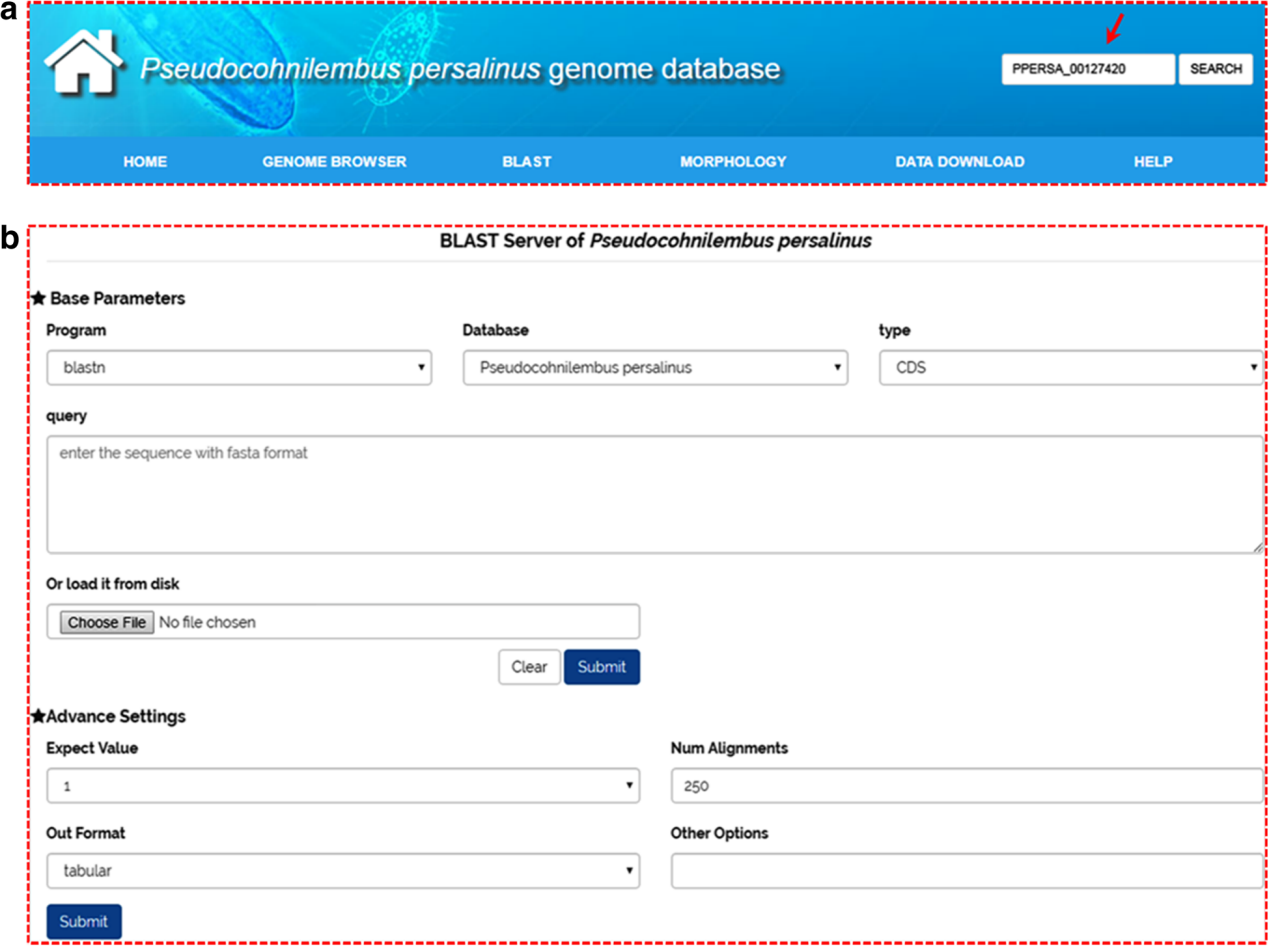
Basic search functions of the PPGD. **a** A basic search function using the “Gene ID,” “Keywords,” or “Scaffold ID”. Red arrow, search box containing the example gene ID, PPERSA_00127420. **b** BLAST search tool

**Fig. 3:**
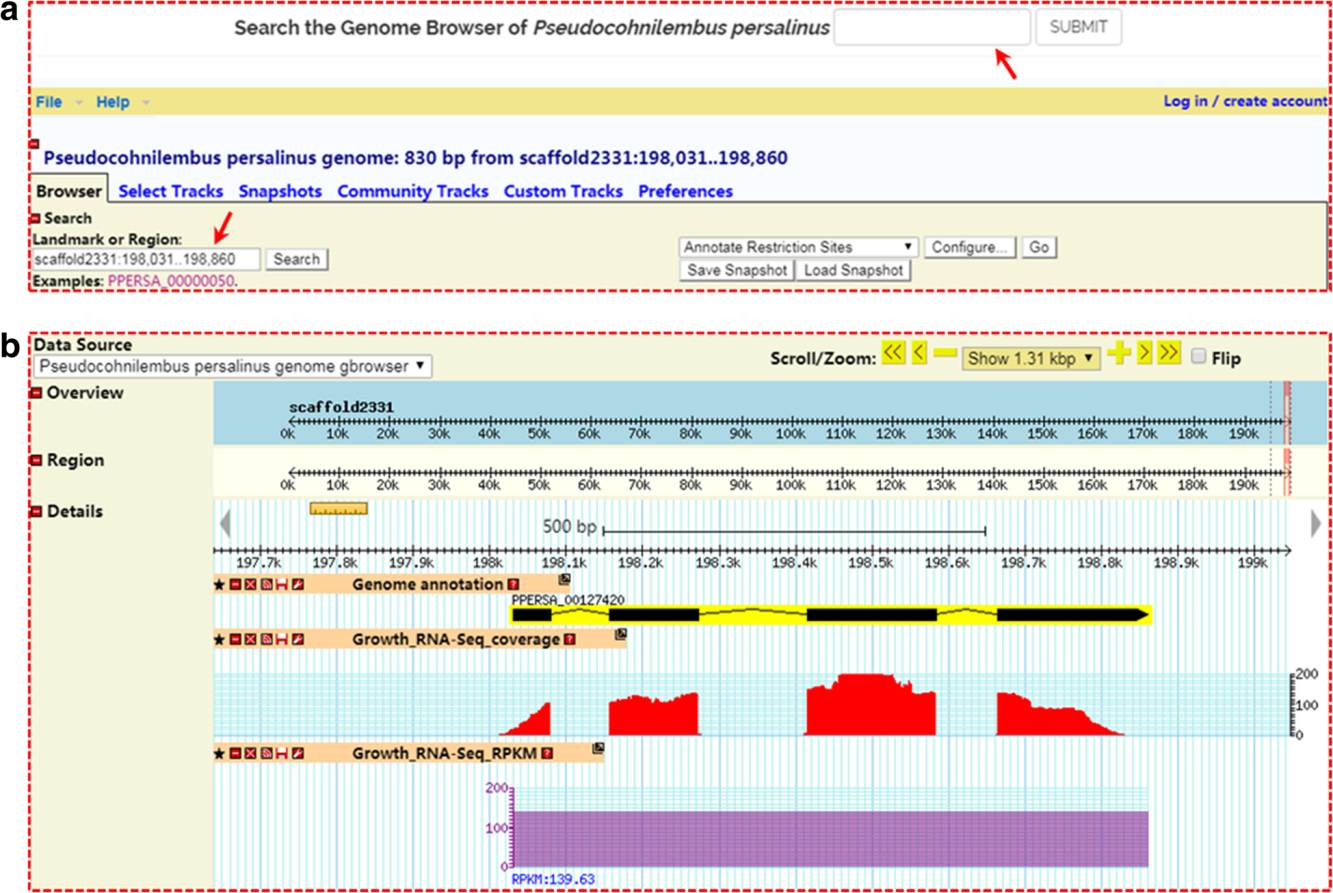
A GBrowse screenshot shows search results for the example gene, PPERSA_00127420. **a** Search function of GBrowse. Red arrow, search box. **b** A case (gene PPERSA_00127420) shows the GBrowse search result with three main tracks: gene structure, RNA-Seq coverage, and expression level (RPKM value)

A search box (located at the top right of the web page; Fig. 2a) enables the user to search for “Gene ID,” “Keywords,” or “Scaffold ID” in the database (Fig. 2a). We recommend searching the database using a “Gene ID”. The search hits are listed and hyperlinks direct the user to detailed information about the gene. This consists of five main sections (Fig. 3): (i) basic information, including the gene name and location (Fig. 3a); (ii) a GBrowse snapshot showing the gene structure (Fig. 3b); (iii) protein domain, protein structure and GO annotation (Fig. 3c, d); (iv) gene information on homolog (Fig. 3e); and (v) coding sequence (CDS) and protein sequence (Fig. 3f).

The PPGD also offers a search function for homologous sequence in BLAST, which is embedded into the database to provide a graphical interface (Fig. 2b). This allows users to search for gene information on homologs by directly inserting a query sequence into the text box. The CDS, protein sequence, and whole draft genome sequence of *P. persalinus* are organized as datasets for BLAST searching. Furthermore, “Expect value” and “Number alignments” options are included as advanced settings to filter low-comparability sequence. Finally, users can select an output format for downloading the homologous sequence.

### Data visualization

**Fig. 4:**
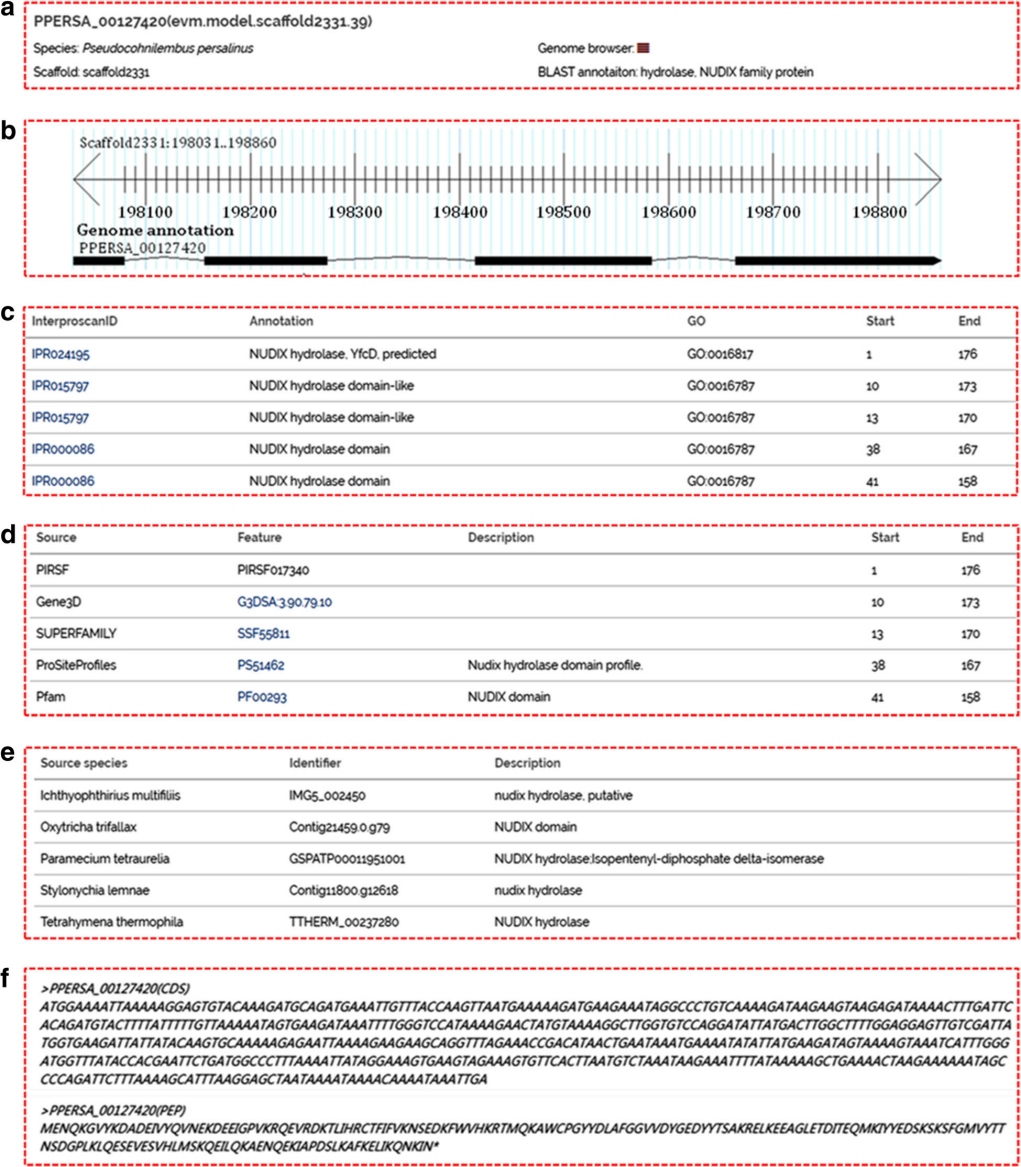
Search result page shows detailed genetic information for the example gene, PPERSA_00127420. **a** Basic description. **b** GBrowse snapshot. **c** InterproScan annotations. (d) Protein domain annotations from various databases. **e** Ortholog information. **f** Sequence information, including predicted CDS and protein sequence

GBrowse has been implemented in PPGD for visualizing gene annotation and transcriptomic data. Typically, the user can use “Gene ID” or a scaffold region to search within GBrowse (Fig. 4a). The GBrowse search will mainly return three tracks: (i) a putative gene model; (ii) an RNA-Seq coverage plot for growth conditions; and (iii) RPKM values (Fig. 4b). GBrowse provides the user with information on the gene structure and expression levels. In the gene model track, a hyperlink forwards the user to a web page with more detailed gene information. In addition, once an interesting gene or genomic region is selected, the user can scroll and zoom using the navigation bar (shown in the upper right of Fig. 4). The sequence can also be retrieved in FASTA format by selecting the “Download Decorated FASTA File” option in the drop-down menu in GBrowse.

### Other functions

The “MORPHOLOGY” page of PPGD displays detailed morphological information and the taxonomic status of *P. persalinus*, based on previous studies [5, 14–18]. Users can also download *P. persalinus* genomic sequence, protein sequence, and CDS in FASTA format and gene annotation information in CSV format via the “DATA DOWNLOAD” page.

### Further development

We will continue to update PPGD by integrating more data, such as functional genomics data. As more and more ciliate genomes are sequenced, we will try to integrate these into PPGD to build a comprehensive genome database for ciliates.

## Conclusions

PPGD is the first genome database for a scuticociliate. It integrates basic genomic information of *P. persalinus* and provides a useful resource for studying scuticociliates and scuticociliatosis.

## Abbreviations

BLAST: Basic Local Alignment Search Tool
CDS: Coding sequence
GBrowse: Generic Genome Browser
GO: Gene Ontology
MAC: Macronucleus
MIC: Micronucleus
NCBI: National Center for Biotechnology Information
PPGD: *Pseudocohnilembus persalinus* genome database
RPKM: Reads per kilobase per million mapped reads
